# Novel Implications of MicroRNAs, Long Non-coding RNAs and Circular RNAs in Drug Resistance of Esophageal Cancer

**DOI:** 10.3389/fcell.2021.764313

**Published:** 2021-11-22

**Authors:** Ling Wei, Jujie Sun, Nasha Zhang, Yue Shen, Teng Wang, Zengjun Li, Ming Yang

**Affiliations:** ^1^Shandong Provincial Key Laboratory of Radiation Oncology, Cancer Research Center, Shandong Cancer Hospital and Institute, Shandong First Medical University and Shandong Academy of Medical Sciences, Jinan, China; ^2^Department of Pathology, Shandong Cancer Hospital and Institute, Jinan, China; ^3^Department of Radiation Oncology, Shandong Cancer Hospital and Institute, Jinan, China; ^4^Department of Endoscopy, Shandong Cancer Hospital and Institute, Jinan, China

**Keywords:** esophageal cancer, drug resistance, microRNA, long non-coding RNA, circular RNA

## Abstract

Esophageal cancer is the eighth most common malignancy and the sixth leading cause of cancer-related deaths worldwide. Chemotherapy based on platinum drugs, 5-fluorouracil, adriamycin, paclitaxel, gemcitabine, and vinorelbine, as well as targeted treatment and immunotherapy with immune checkpoint inhibitors improved the prognosis in a portion of patients with advanced esophageal cancer. Unfortunately, a number of esophageal cancer patients develop drug resistance, resulting in poor outcomes. Multiple mechanisms contributing to drug resistance of esophageal cancer have been reported. Notably, non-coding RNAs (ncRNAs), including microRNAs (miRNAs), long non-coding RNAs (lncRNAs) and circular RNAs (circRNAs), have been identified to play crucial roles in modulating esophageal cancer drug resistance. In the present review, we highlight the underlying mechanisms how miRNAs, lncRNAs, and circRNAs impact the drug resistance of esophageal cancer. Several miRNAs, lncRNAs, and circRNAs may have potential clinical implications as novel biomarkers and therapeutic targets for esophageal cancer.

## Introduction

Esophageal cancer is a complex malignancy and the sixth leading cause of cancer death worldwide. A total of 572,034 esophageal cancer cases were diagnosed worldwide and 508,585 cases dead in 2018 (Bray et al., 2018). There are two major histological subtypes of esophageal cancer, esophageal adenocarcinoma (EAC) and esophageal squamous cell carcinoma (ESCC). EAC is prevalent in Western countries including North America and Western Europe; while ESCC is the major histologic type of esophageal cancer in Eastern Asia and Africa (Bray et al., 2018). In the past 15 years, the 5-year survival rate for patients with esophageal cancer for all stages combined is only 20%. However, patients with metastatic esophageal cancer have a poor prognosis, with the 5-year survival rate of approximately 5% (Bray et al., 2018; Siegel et al., 2020).

Although treatment options are limited for patients with unresectable, locally advanced, or metastatic esophageal cancer, a portion of patients could benefit from the comprehensive treatment of chemotherapy, targeted therapy and immunotherapy with immune checkpoint inhibitors (Eltweri et al., 2019; Kojima et al., 2020; Lopez et al., 2020; Stroes et al., 2020). The commonly used chemotherapeutic agents in clinics include platinum drugs, 5-fluorouracil (5-FU), adriamycin (ADM), paclitaxel (PTX), irinotecan, gemcitabine (GEM), and vinorelbine (Liu S. L. et al., 2015; Wang Y. S. et al., 2016; Tin et al., 2018; van Zweeden et al., 2018; Ni et al., 2020). Molecular targeted therapy drugs include anti-HER2 monoclonal antibodies (trastuzumab and pertuzumab), oral tyrosine kinase inhibitors (TKIs) targeting HER-1/HER-2 (lapatinib), anti-vascular endothelial growth factor receptor 2 (VEGFR-2) antibody (ramucirumab) and anti-EGFR monoclonal antibody (panitumumab) (Press et al., 2017; Shepard et al., 2017; Chakrabarti et al., 2018; Guo et al., 2018; Yamaguchi et al., 2018; De Vita et al., 2019; Hassan et al., 2019; Rogers et al., 2019; Wagner et al., 2019; Stroes et al., 2020). Immune-checkpoint blockade agents are also used, such as anti-programmed cell death protein 1 (PD-1) monoclonal antibodies (nivolumab and pembrolizumab) (Herbst et al., 2019; Shah et al., 2019; Kato et al., 2020; Rogers et al., 2020).

Unfortunately, esophageal cancer cells frequently develop multi-drug resistance (MDR) which seriously impaired the efficacy of drugs and subsequently led to poor prognosis. The underlying complicated mechanisms involved in drug resistance of esophageal cancer have been reported, such as the enhanced DNA damage repair capability, the up-regulated expression of drug efflux transporters to pump out chemo-agents from cells, the accelerated cell growth and autophagy flux, dysregulation of cell cycle, epithelial-mesenchymal transition (EMT), apoptosis inactivation as well as activation of cancer stem cells (CSCs) (Liu D. S. et al., 2015; Cheng et al., 2017; Zhou et al., 2017, 2020; Huang et al., 2018; Qiao et al., 2018; Guo et al., 2019; Lin C. H. et al., 2019).

**FIGURE 1:**
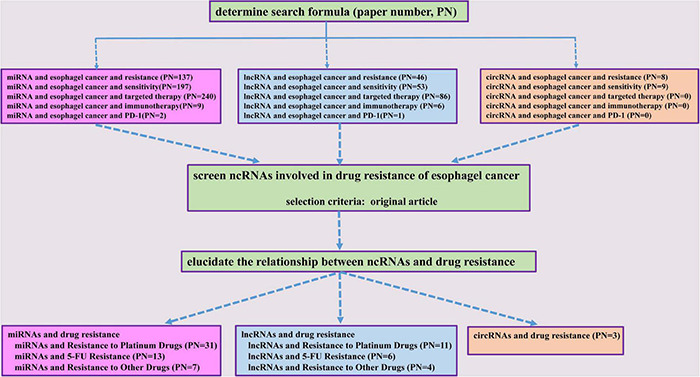
A flow diagram of the study selection process.

Non-coding RNAs (ncRNAs) are a class of RNA transcripts without protein-coding ability, such as microRNAs (miRNAs), long non-coding RNAs (lncRNAs), and circular RNAs (circRNAs). Multiple miRNAs, lncRNAs, and circRNAs have been reported to be involved in controlling various cellular functions, such as apoptosis, cell growth, autophagy, EMT, and cell cycle regulation (Wang et al., 2015c; Bhan et al., 2017; Peng et al., 2017; Ren et al., 2017a, b; Rupaimoole and Slack, 2017; Pan et al., 2018; Vo et al., 2019; Chen et al., 2020; Yuan et al., 2020; Zhang et al., 2020a). Among these ncRNAs, several miRNAs, lncRNAs, and circRNAs are dysregulated in esophageal cancer and have been shown to be associated with tumorigenesis, metastasis, prognosis, as well as treatment resistance to radiotherapy and drugs (Song et al., 2014; Wang et al., 2015b; Wen et al., 2016; Zhang E. et al., 2017; Zhang et al., 2018; Sang et al., 2018). So far, there have been few reports of ncRNAs involvement in the resistance to targeted therapy and immunotherapy. Interestingly, serum miRNAs, including miR-1233-5p, miR-6885-5p, miR-4698, and miR-128-2-5p, have been identified to predict the response to nivolumab, a PD-1 inhibitor, in advanced ESCC patients (Sudo et al., 2020). Notably, a number of miRNAs, lncRNAs, and circRNAs have been shown to play crucial roles in esophageal cancer chemoresistance (Jin et al., 2016; Li et al., 2018; Lin K. et al., 2019; Wu et al., 2020; Zou et al., 2020). Considering the importance of ncRNAs in the development of drug resistance of esophageal cancer, we systematically summarized the underlined mechanisms of these miRNAs, lncRNAs, and circRNAs in the current review (Figure 1).

## MicroRNAs and Drug Resistance

MicroRNAs are small, endogenous, single-stranded ncRNAs and function as crucial regulators of gene expression at the post-transcriptional level. MiRNAs down-regulate expression levels of target genes through binding to the 3′-untranslated region (3′-UTR) of target mRNA and leading to target mRNA degradation or blocking translation. It has been found that miRNAs can act as oncogenes or tumor suppressors to regulate cell differentiation, proliferation, apoptosis, metabolic reprogramming and angiogenesis (Bracken et al., 2016; Rupaimoole and Slack, 2017). Additionally, miRNAs could be released from esophageal cancer cells via exosomes and affect neighboring or distant cells. The exchange of the genetic information and/or regulation of target gene expression of miRNAs may change biological behaviors of recipient cells (Tanaka Y. et al., 2013; Luo et al., 2019; Gao et al., 2020). Multiple aberrantly expressed miRNAs have been identified in esophageal cancer, especially in the development of drug resistance (Hamano et al., 2011; Wang Y. et al., 2016; Liu et al., 2017; Zhang J. X. et al., 2017). Here, we summarized the roles of miRNAs in the resistance to platinum drugs, 5-FU and other agents in esophageal cancer.

### MicroRNAs and Resistance to Platinum Drugs

Platinum drugs are the most commonly used antitumor drugs in clinic. In cells, platinum binds to genomic DNA to form platinum-DNA adducts, resulting in DNA replication and transcription disorders, and subsequently tumor cell death. Multiple platinum drugs have been applied in clinical managements of esophageal cancer, such as cisplatin (DDP, the first-generation platinum agent), carboplatin (the second-generation platinum agent), oxaliplatin, and loplatin (the third-generation platinum agents). However, response rates to platinum drugs are low in some esophageal cancer patients. Several miRNAs have been reported to participate in development of resistance to platinum drugs in esophageal cancer (Table 1).

**TABLE 1:** MicroRNAs (miRNAs) and platinum drugs resistance in esophageal cancer.

**MiRNAs**	**Expression^a^**	**Genes and pathways**	**Drug**	**References**
miR-10b	↑	PPARγ/AKT/mTOR/P70S6K	Cisplatin	Wu et al., 2020
miR-432-3p	↑	KEAP1/NRF2	Cisplatin	Akdemir et al., 2017
miR-141	↑	YAP1	Cisplatin	Imanaka et al., 2011
miR-200c	↑	PPP2R1B/AKT	Cisplatin	Hamano et al., 2011
miR-21	↑	–	Cisplatin	Komatsu et al., 2016a
		PDCD4	Cisplatin	Yang et al., 2019
miR-27a/b	↑	CAF	Cisplatin	Tanaka et al., 2015
miR-483	↑	–	Cisplatin	Zhou and Hong, 2013
miR-214	↑	–	Cisplatin	Zhou and Hong, 2013
miR-223	↑	PARP	Cisplatin	Streppel et al., 2013
miR-196a	↑	ABCG2	Cisplatin	Ma et al., 2016
miR-296	↑	P-gp, Bcl-2, Bax, cyclin D1, P27	Cisplatin	Hong et al., 2010
miR-455-3p	↑	Wnt/β-catenin	Cisplatin	Liu et al., 2017
miR-193	↑	TFAP2C, cyclin D1, bax, caspase 3	Cisplatin	Shi et al., 2020
miR-106b-3p	↑	TGM3	Cisplatin	Zhu Y. et al., 2021
miR-141-3p	↑	PTEN	Oxaliplatin	Jin et al., 2016
miR-544	↓	E2F5	Cisplatin	Sun et al., 2019
miR-338-5p	↓	FERMT2	Cisplatin	Lin W. C. et al., 2019
miR-125a-5p	↓	STAT3	Cisplatin	Zhao et al., 2018
miR-218	↓	Survivin	Cisplatin	Jingjing et al., 2016
		PI3K/AKT/mTOR	Cisplatin	Tian et al., 2015
miR-214-3p	↓	CUG-BP1, survivin	Cisplatin	Phatak et al., 2016
miR-499	↓	polβ	Cisplatin	Wang et al., 2015d
let-7c	↓	IL-6/STAT3	Cisplatin	Sugimura et al., 2012
let-7g/i	↓	ABCC10	Oxaliplatin	Wu et al., 2016
miR-634	↓	OPA1, TFAM, LAMP2, APIP, XIAP, BIRC5, NRF2	Cisplatin	Fujiwara et al., 2015
miR-187	↓	C3	Cisplatin	Winther et al., 2016
miR-130a-3p	↓	Bcl-2	Cisplatin	Lindner et al., 2018
miR-145	↓	PI3K/AKT, MRP1, P-gp	Cisplatin	Zheng et al., 2019
miR-181a-5p	↓	CBLB	Cisplatin	Yang S. et al., 2020
miR-153-3p	↓	Nrf-2	Cisplatin	Zuo et al., 2020

*^*a*^miRNAs either up-regulated (↑) or down-regulated (↓) in platinum drugs resistant esophageal cancer cells. This table shows 29 miRNAs whose expression levels and potential targets in platinum drugs resistance of esophageal cancer.*

There are many oncogenic miRNAs promoting the resistance of esophageal cancer to platinum drugs (Table 1). MiR-10b could enhance DDP resistance through silencing peroxisome proliferator-activated receptor-γ (PPARγ) and activating the AKT/mTOR/p70S6K signaling pathway (Wu et al., 2020). MiR-432-3p has been found to promote the resistance to DDP by directly suppressing expression of Kelch-like ECH-associated protein 1 (KEAP1) and stabilizing NF-E2-related factor 2 (NRF2). On the contrary, miR-432-3p knocking-off through the CRISPR/Cas9 technology could reverse DDP resistance of ESCC cells (Akdemir et al., 2017). As the most highly expressed miRNA in DDP-resistant ESCC cells, miR-141 could potentiate the resistance to DDP by directly silencing Yes-associated protein 1 (YAP1) (Imanaka et al., 2011). In addition, the expression of miR-200c was also found to be significantly up-regulated in DDP-resistant esophageal cancer cells compared to their parent cells. Mechanistically, miR-200c increases DDP resistance through modulating activity of the AKT pathway (Hamano et al., 2011). Among esophageal cancer patients, miR-200c levels were markedly correlated with response to chemotherapy. That is, high levels of miR-200c in patient serum were significantly correlated with poor response to neoadjuvant chemotherapy treated with DDP, 5-FU and adriamycin (ACF) (Tanaka K. et al., 2013). Ectopic miR-21 in ESCC cells has been found to promote DDP resistance (Komatsu et al., 2016a). Moreover, the levels of miR-21 and miR-23a in pre-operative plasma of ESCC patients might be used to predict the resistance to pre-operative chemotherapy regimens with DDP plus 5-FU (Komatsu et al., 2016a, b). Consistently, exosome-derived oncogenic miR-21 has also been shown to weaken DDP sensitivity of esophageal cancer cells by silencing programmed cell death 4 (PDCD4) (Yang et al., 2019). Interestingly, miR-27a/b may confer DDP resistance through transforming normal fibroblast into cancer-associated fibroblasts (CAF). Although ectopic miR-27a/b could not significantly impair chemosensitivity of esophageal cancer cells, the supernatant originating from miR-27a/b-transfected CAFs has been shown to promote DDP resistance in esophageal cancer cells, compared with supernatant deriving from normal fibroblast. Moreover, the resistance to DDP could be overcame after adding neutralized antibody against transforming growth factor-β (TGF-β) to the supernatant (Tanaka et al., 2015). MiR-483 and miR-214 were dramatically up-regulated in ESCC tissues compared with those in normal tissues and could confer DDP resistance in ESCC cells (Zhou and Hong, 2013). Oncogenic miR-223 has been found to diminish DNA repair and apoptosis potentials of esophageal cancer cells and increase the resistance to DDP via targeting and down-regulating Poly (ADP-ribose) polymerase 1 (PARP1) (Streppel et al., 2013). MiR-196a and miR-296 could promote DDP resistance via promoting the expression of cell membrane transporter ATP binding cassette subfamily G member 2 (ABCG2) and P-glycoprotein (P-gp) (Hong et al., 2010; Ma et al., 2016). Oncogenic miR-455-3p could increase the subpopulations of CD90^+^ and CD271^+^ CSCs/tumor-initiating cells (T-ICs) through activating the Wnt/β-catenin signaling and the TGF-β signaling, which leads to resistance of ESCC cells to DDP (Liu et al., 2017). MiR-193, a highly expressed miRNA in DDP-resistant esophageal cancer cell exosomes (TE-1/DDP/exo), has been shown to promote DDP resistance by targeting transcription factor AP-2 gamma (TFAP2C). Moreover, level of high miR-193 or low TFAP2C could suppress apoptosis and abate cell cycle inhibition (Shi et al., 2020). Most recently, miR-106b-3p, an overexpressed miRNA in ESCC tissues, has also been demonstrated to confer the resistance to DDP by targeting glutamine γ-glutamyltransferase E (TGM3) in esophageal cancer cells (Zhu Y. et al., 2021). In addition, oncogenic miR-141-3p is highly expressed in oxaliplatin-resistant esophageal cancer cells and has been found to enhance resistance by silencing phosphatase and tensin homolog (PTEN), *in vitro* and *in vivo* (Jin et al., 2016).

By contrast, several tumor suppressor miRNAs can reverse resistance of esophageal cancer to platinum drugs (Table 1). Ectopic miR-544 and miR-338-5p could overcome DDP resistance of esophageal cancer via targeting and down-regulating oncogene E2F transcription factor 5 (E2F5) and fermitin family homolog 2 (FERMT2) (Lin W. C. et al., 2019; Sun et al., 2019). Similarly, ectopic miR-125a-5p could potentiate the cytotoxic and apoptotic effects of DDP on esophageal cancer cells through modulating the signal transducer and activator of transcription 3 (STAT3) signaling pathway (Zhao et al., 2018). Tumor suppressor miR-218 could reverse DDP resistance and promote apoptosis of esophageal cancer cells by silencing oncogene survivin (Jingjing et al., 2016). Interestingly, miR-218 could inhibit cell proliferation, promote cell apoptosis, induce cell cycle arrested in G_0_/G_1_ phase, as well as increase DDP sensitivity of esophageal cancer cells through suppressing phosphorylation of PI3K, AKT, and mTOR (Tian et al., 2015). MiR-214-3p, a highly down-regulated miRNA in ESCC cells, could weaken DDP resistance by targeting and down-regulating both survivin and RNA-binding protein (RBP) CUG-BP1 (Phatak et al., 2016). Tumor suppressor miR-499 have also been found to reverse the DDP resistance of esophageal cancer cells by silencing DNA polymerase β (polβ) (Wang et al., 2015d). Through suppressing the IL-6/STAT3 pathway and drug transporter ABCC10, let-7, and let-7g/i could restore the sensitivity to DDP and oxaliplatin and promote apoptosis of esophageal cancer cells (Sugimura et al., 2012; Wu et al., 2016). Tumor suppressor miR-634 could enhance the cytotoxicity induced by DDP via concurrently targeting multiple genes which were linked with anti-apoptosis, mitochondrial homeostasis, autophagy and antioxidant ability. Specifically, anti-apoptotic genes include APAF1 interacting protein (APIP), baculoviral IAP repeat containing 5 (BIRC5), and E3 ubiquitin protein ligase X-linked inhibitor of apoptosis (XIAP); mitochondrial homeostasis genes involve transcription factor A, mitochondrial (TFAM) and optic atrophy 1 (OPA1); autophagy and antioxidant genes refer to lysosomal-associated membrane protein 2 (LAMP2) and NRF2 (NFE2L2; nuclear factor, erythroid 2-like 2) (Fujiwara et al., 2015). MiR-187 was significantly down-regulated in pre-treatment tumors of EAC patients with worse response to neoadjuvant chemoradiation therapy. Mechanistically, miR-187 could reverse the resistance to DDP and X-ray irradiation in EAC cells by modulating multiple signaling pathways, including the complement component 3 (C3) signaling (Winther et al., 2016). Through silencing Bcl-2, miR-130a-3p could sensitize esophageal cancer cells to DDP (Lindner et al., 2018). By suppressing the PI3K/AKT pathway and expression of MDR-associated proteins MRP1 and P-gp, tumor suppressor miR-145 could sensitize ESCC to DDP and promote DDP-induced apoptosis and cell cycle arrest (Zheng et al., 2019). Most recently, miR-181a-5p, a down-regulated miRNA in DDP-resistant EAC cell line (OE19/DDP), has also been demonstrated to reverse the resistance to DDP in EAC by modulating CBLB. Moreover, ectopic expression of miR-181a-5p could potentiate the *in vivo* sensitivity to DDP in EAC (Yang S. et al., 2020). Additionally, tumor suppressor miR-153-3p could also potentiate the sensitivity of EC cells to DDP via Nrf-2 (Zuo et al., 2020).

### MicroRNAs and 5-Fluorouracil Resistance

5-fluorouracil is a heterocyclic aromatic chemotherapeutic agent which is broadly utilized in esophageal cancer treatments. 5-FU inhibits thymidylate synthase (TS), hampers DNA replication, and subsequently resulting in arrested cell cycle and apoptosis (Longley et al., 2003; Subbarayan et al., 2010). It has been reported that several oncogenic or tumor suppressive miRNAs are involved in 5-FU resistance (Table 2).

**TABLE 2:** MicroRNAs (miRNAs) and 5-FU resistance in esophageal cancer.

**MiRNAs**	**Expression^a^**	**Genes and pathways**	**References**
miR-141-3p	↑	PTEN	Jin et al., 2016
miR-221	↑	DKK2	Wang Y. et al., 2016
miR-21	↑	–	Komatsu et al., 2016a
miR-214	↑	–	Zhou and Hong, 2013
miR-483	↑	–	Zhou and Hong, 2013
miR-193a-3p	↑	PSEN1	Meng et al., 2016
miR-193b-3p	↑	KRAS	Hummel et al., 2014
miR-27b-3p	↑	KRAS	Hummel et al., 2014
miR-296	↑	P-gp, Bcl-2, Bax, cyclin D1, P27	Hong et al., 2010
miR-193b	↓	Stathmin 1	Nyhan et al., 2016
miR-634	↓	OPA1, TFAM, LAMP2, APIP, XIAP, BIRC5, NRF2	Fujiwara et al., 2015
miR-192-5p	↓	TYMS	Hummel et al., 2014
miR-378a-3p	↓	CBL-B	Hummel et al., 2014
miR-194-5p	↓	ABCC3	Hummel et al., 2014
miR-18a-3p	↓	KRAS	Hummel et al., 2014
miR-125a-5p	↓	ERBB2	Hummel et al., 2014
miR-145	↓	REV3L	Chen Q. et al., 2019
miR-29c	↓	FBXO31	Li et al., 2019
miR-338-5p	↓	Id-1	Han et al., 2019

*^*a*^miRNAs either up-regulated (↑) or down-regulated (↓) in 5-FU resistant esophageal cancer cells. This table shows 19 miRNAs whose expression levels and potential targets in 5-FU resistance of esophageal cancer.*

Multiple oncogenic miRNAs could promote 5-FU resistance of esophageal cancer cells. Oncogenic miR-141-3p can confer 5-FU resistance by silencing PTEN and the elevated levels of miR-141-3p was associated with TNM stage and differentiation status of ESCC patients (Jin et al., 2016). MiR-221 was overexpressed in 5-FU resistant esophageal cancer cells and EAC tissue and could potentiate 5-FU resistance by directly down-regulating the expression of dickkopf Wnt signaling pathway inhibitor 2 (DKK2) and activating the Wnt/β-catenin-EMT pathways (Wang Y. et al., 2016). In 5-FU resistant esophageal cancer cells, miR-27b-3p and miR-193b-3p have been found to be significantly up-regulated. Ectopic miR-27b-3p and miR-193b-3p could promote 5-FU resistance though silencing expression of their target gene KRAS (Hummel et al., 2014). MiR-296 has been found to contribute to 5-FU resistance in esophageal cancer cells through modulating the expression of P-gp, Bcl-2, Bax, cyclin D1 and P27 (Hong et al., 2010). In addition, oncogenic miR-21, miR-214, and miR-483 could also promote 5-FU resistance of ESCC cells (Zhou and Hong, 2013; Komatsu et al., 2016a). By targeting presenilin-1 (PSEN1), miR-193a-3p could also confer 5-FU resistance of esophageal cancer cells (Meng et al., 2016).

On the contrary, a number of tumor suppressor miRNAs can reverse 5-FU resistance of esophageal cancer cells. MiR-193b was highly expressed in chemosensitive esophageal cancer cells. MiR-193b has been shown to significantly promote the sensitivity to 5-FU in KYSE450 cells by silencing stathmin 1, which leads to activation of the autophagic flux and non-apoptotic cell death (Nyhan et al., 2016). Additionally, tumor suppressor miR-634 have also been found to be involved in development of 5-FU resistance by directly targeting a number of mitochondrial apoptosis pathway genes, such as *OPA1*, *TFAM*, *LAMP2*, *APIP*, *XIAP*, *BIRC5*, and *NRF2* (Fujiwara et al., 2015). Several dysregulated miRNAs, including miR-192-5p, miR-378a-3p, miR-194-5p, miR-18a-3p, and miR-125a-5p, have been identified to be down-regulated in 5-FU resistant esophageal cancer cells. Ectopic miR-192-5p, miR-378a-3p, miR-194-5p, miR-18a-3p, and miR-125a-5p could reverse 5-FU resistance through silencing the expression of their target genes thymidylate synthase (TYMS), CBL-B, ABCC3, KRAS, and ERBB2 (Hummel et al., 2014). In ESCC cells treated with 5-FU, miR-145 has been found to obviously enhance apoptosis and expression of Bax, Bcl-2, and caspase3, via down-regulating REV3L (Chen Q. et al., 2019). MiR-29c was down-regulated in tumor tissues and serum samples of ESCC patients and has also been found to reverse 5-FU resistance by silencing F-box only protein 31 (FBXO31) (Li et al., 2019). Tumor suppressor miR-338-5p was down-regulated in 5-FU resistant ESCC cells as well as sera and tumor tissue of ESCC patients. Low miR-338-5p levels in serum was associated with poor response to neoadjuvant chemoradiotherapy based on 5-FU/DDP and worse survival of ESCC patients. Mechanistically, miR-338-5p could restore 5-FU sensitivity of ESCC cells by silencing the gene expression of inhibitor of differentiation 1 (Id-1) *in vitro* and *in vivo* (Han et al., 2019).

### MicroRNAs and Resistance to Other Drugs

In clinics, adriamycin, vincristine, paclitaxel, gemcitabine, and vinorelbine are also commonly used in esophageal cancer therapy. Multiple miRNAs have been shown to participate in their resistance (Table 3). MiR-27a could confer adriamycin resistance and inhibit the apoptosis induced by adriamycin. It has been found that miR-27a could increase the expression of P-gp and Bcl-2, as well as reduce Bax expression in esophageal cancer cells (Zhang et al., 2010). Besides promoting the resistance to DDP and 5-FU, miR-483 and miR-214 could also potentiate adriamycin resistance and reduce intracellular accumulation of adriamycin in esophageal cancer cells (Zhou and Hong, 2013). MiR-223 has also been found to confer adriamycin resistance through inhibiting PARP levels (Streppel et al., 2013). Oncogenic miR-296 could confer the resistance to adriamycin and vincristine through silencing gene expression controlling apoptosis and cell cycle (Hong et al., 2010). For paclitaxel resistance, it has been reported that the combined miR-133a and miR-133b down-regulation could predict the sensitivity to paclitaxel-based chemotherapy in ESCC patients (Chen et al., 2014). Interestingly, esophageal cancer patients with low expression of miR-214 appeared to show higher sensitivity to the combination regimen of gemcitabine plus vinorelbine, indicating that miR-214 may predict esophageal cancer chemosensitivity (Wang Y. S. et al., 2016). In addition, miR-193a-3p has been found to potentiate the chemoresistance to docetaxel, paclitaxel and vinorelbine in esophageal cancer cells via silencing PSEN1 (Meng et al., 2016).

**TABLE 3:** MicroRNAs (miRNAs) and resistance to other drugs in esophageal cancer.

**MiRNAs**	**Expression^a^**	**Genes and pathways**	**Drugs**	**References**
miR-27a	↑	MDR1, Bcl-2, Bax	ADM	Zhang et al., 2010
miR-483	↑	–	ADM	Zhou and Hong, 2013
miR-214	↑	–	ADM	Zhou and Hong, 2013
miR-223	↑	PARP	ADM	Streppel et al., 2013
miR-296	↑	P-gp, Bcl-2, Bax, cyclinD1, P27	ADM, vincristine	Hong et al., 2010
miR-133a/b	↑	–	PTX	Chen et al., 2014
miR-214	↑	–	GEM, vinorelbine	Wang Y. S. et al., 2016
miR-193a-3p	↑	PSEN1	Docetaxel, PTX, vinorelbine	Meng et al., 2016

*^*a*^miRNAs up-regulated (↑) in other drugs resistant esophageal cancer cells. This table shows eight miRNAs whose expression levels and potential targets in other drugs resistance of esophageal cancer. ADM, adriamycin; PTX, paclitaxel; GEM, gemcitabine.*

## Long Non-Coding RNAs and Drug Resistance

Long non-coding RNAs are a group of ncRNAs longer than 200 nt without protein-coding capacity. Accumulating evidences showed that lncRNAs play important roles in regulating various cellular processes (Frye et al., 2016; Kopp and Mendell, 2018). For instance, lncRNAs could regulate target gene expression at either the transcriptional level or the post-transcriptional level through interaction with various DNA, RNA or proteins. Abnormally expressed lncRNAs have been identified in almost all cancer types, including esophageal cancer (Li J. et al., 2014; Yang et al., 2014; Tan et al., 2017; Xu et al., 2019) and have been implicated in diagnosis, metastasis, prognosis, radioresistance, and chemoresistance of esophageal cancer (Li W. et al., 2014; Wang et al., 2015a, 2017; Lin et al., 2018; You et al., 2019; Zhang H. et al., 2019; Liu J. et al., 2020). Importantly, several lncRNAs have been found to contribute to development of drug resistance in esophageal cancer.

### Long Non-coding RNAs and Resistance to Platinum Drugs

It has been found that multiple lncRNAs were involved in the resistance to platinum drugs in esophageal cancer (Table 4), including oncogenic lncRNAs NSUN2 methylated lncRNA (NMR), colon cancer-associated transcript-1 (CCAT1), taurine up-regulated gene 1 (TUG1), TP73-AS1, prostate cancer associated ncRNA transcript 1 (PCAT-1), AFAP1-AS1, FOXD2-AS1, POU3F3, LINC00337, LINC00152, and tumor suppressive lncRNA tumor suppressor candidate 7 (TUSC7).

**TABLE 4:** Long non-coding RNAs (lncRNAs) and platinum drugs resistance in esophageal cancer.

**LncRNAs**	**Expression^a^**	**Genes and pathways**	**Drugs**	**References**
NMR	↑	BPTF	Cisplatin	Li et al., 2018
CCAT1	↑	miR-143/PLK1/BUBR1	Cisplatin	Hu M. et al., 2019
TUG1	↑	Nrf2	Cisplatin	Zhang Z. et al., 2019
TP73-AS1	↑	–	Cisplatin	Zang et al., 2016
PCAT-1	↑	–	Cisplatin	Zhen et al., 2018
AFAP1-AS1	↑	–	Cisplatin	Zhou et al., 2016
FOXD2-AS1	↑	miR-195/Akt/mTOR	Cisplatin	Liu H. et al., 2020
POU3F3	↑	IL-6	Cisplatin	Tong et al., 2020
LINC00337	↑	TPX2, E2F4	Cisplatin	Yang C. et al., 2020
LINC00152	↑	ZEB1, EZH2	Oxaliplatin	Zhang et al., 2020b
TUSC7	↓	miR-224/DESC1/EGFR/AKT	Cisplatin	Chang et al., 2018

*^*a*^lncRNAs either up-regulated (↑) or down-regulated (↓) in platinum drugs resistant esophageal cancer cells. This table shows 11 lncRNAs whose expression levels and underlying pathways in platinum drugs’ resistance of esophageal cancer.*

Oncogenic lncRNA NMR (namely ENST00000432429.1 in GENCODE v13 or ENST00000432429.5 in Ensembl release 83), highly methylated by methyltransferase NSUN2 which can catalyze cytosine methylation to 5-methylcytosine (m5C) in tRNA and some poly(A) RNAs, has been found to play crucial roles in regulating DDP resistance and metastasis of ESCC cells (Li et al., 2018). LncRNA NMR was evidently up-regulated in ESCC and associated with poor overall survival (OS) of ESCC patients (Li et al., 2018). Functionally, ectopic expression of lncRNA NMR could not only suppress DDP-induced apoptosis, but also promote invasion and migration of ESCC cells. Mechanistically, it has been shown that lncRNA NMR could competitively suppress potential mRNAs m5C levels, such as procollagen-lysine, 2-oxoglutarate 5-dioxygenase 3 (PLOD3), collagen type IV Alpha 5 (COL4A5), laminin beta 1 (LAMB1), and heparan sulfate proteoglycan 2 (HSPG2). Moreover, lncRNA NMR directly bond to chromatin regulator of bromodomain PHD finger transcription factor (BPTF), and regulated the expression of matrix metallopeptidase 3 (MMP3) and matrix metallopeptidase 10 (MMP10) through the ERK1/2 pathway (Li et al., 2018).

Long non-coding RNA CCAT1, which is highly expressed in esophageal cancer, has also been found to confer DDP resistance in ESCC cells through the miR-143/PLK1/BUBR1 signaling axis (Hu M. et al., 2019). Specifically, silencing of CCAT1 could dramatically potentiate miR-143 expression in a negative regulatory manner and inhibit both mRNA and protein expression of Polo-like kinase 1 (PLK1) and BUBR. Moreover, ectopic expression of miR-143 has been shown to suppress the expression of PLK1, BUBR1, and CCAT1. Functionally, silencing of lncRNA CCAT1 and ectopic miR-143 could reverse DDP drug resistance and inhibit ESCC cell proliferation. Inhibition of lncRNA CCAT1 has also been found to enhance sensitivity of ESCC xenografts in nude mice to DDP, indicating that lncRNA CCAT1 may act as a potential regulator of DDP chemoresistance in esophageal cancer (Hu M. et al., 2019).

Long non-coding RNA TUG1 has also been found to be abundantly expressed in TE-1-derived DDP-resistant esophageal cancer cells TE-1/DDP (Zhang Z. et al., 2019). Mechanistically, lncRNA TUG1 could confer DDP resistance of ESCC cells through elevating P-gp expression and inhibiting apoptosis. Conversely, silencing of lncRNA TUG1 reversed DDP resistance of ESCC cells (Zhang Z. et al., 2019). RNA immunoprecipitation and RNA pull-down assays verified that TUG1 could directly bind the protein of nuclear factor (erythroid-derived 2)-like 2 (Nrf2) and increase Nrf2 protein level. Moreover, Nrf2 antibody could relieve DDP resistance mediated by TUG1 overexpression in ESCC cell, indicating an involvement of TUG1/Nrf2 signaling pathway in DDP resistance (Zhang Z. et al., 2019).

Long non-coding RNAs TP73-AS1 also could promote DDP resistance of esophageal cancer cells (Zang et al., 2016). LncRNA PCAT-1 has been shown to accelerate DDP resistance and tumor growth of esophageal cancer cells (Zhen et al., 2018). Additionally, AFAP1-AS1, a dramatically up-regulated lncRNA in esophageal cancer tissues and DDP-resistant esophageal cancer cells, has been found to be positively associated with not only advanced clinical stages and definitive chemoradiotherapy (dCRT) response, but also shorter OS and progression free survival (PFS) (Zhou et al., 2016). Oncogenic lncRNAs FOXD2-AS1, POU3F3, and LINC00337 were revealed to be involved in DDP resistance of esophageal cancer (Liu H. et al., 2020; Tong et al., 2020; Yang C. et al., 2020). Via the miR-195/Akt/mTOR axis, ectopic expression of FOXD2-AS1, an up-regulated lncRNA in ESCC patients and DDP resistant ESCC cells (TE-1/DDP), could contribute to DDP resistance in ESCC (Liu H. et al., 2020). LncRNA POU3F3 could confer DDP resistance of ESCC cells through exosome POU3F3 inducing normal fibroblasts (NFs) to differentiate into CAFs via secreting interleukin 6 (IL-6). In addition, higher expression of plasma exosome POU3F3 has been shown to predict bad complete response and survival of ESCC patients (Tong et al., 2020). By increasing ESCC cell autophagy, exogenous expression of LINC00337 has been demonstrated to potentially promote DDP resistance through TPX2 up-regulation via recruiting E2F4 (Yang C. et al., 2020). Additionally, through interacting with EZH2, oncogenic LINC00152 has been found to increase ZEB1 expression and accelerate EMT and oxaliplatin resistance in esophageal cancer (Zhang et al., 2020b).

On the contrary, tumor suppressor lncRNA may reverse the resistance of cancer cells to platinum drugs. For instance, lncRNA TUSC7 could overcome the resistance to DDP and promote apoptosis of ESCC cells, via inhibiting miR-224 to modulate differentially expressed in squamous cell carcinoma 1 (DESC1)/EGFR/AKT signaling pathway. Overexpression of DESC1 could reverse the resistance to DDP through EGFR/AKT pathway in ESCC EC9706 and KYSE30 cells. Moreover, esophageal cancer patients with lower lncRNA TUSC7 expression had short OS (Chang et al., 2018).

### Long Non-coding RNAs and 5-Fluorouracil Resistance

Several oncogenic or tumor suppressive lncRNAs are associated with the resistance to 5-FU in esophageal cancer (Table 5). Oncogenic LINC01419 has been found to promote 5-FU resistance and inhibit apoptosis of ESCC cells (Chen J. L. et al., 2019). LINC01419 could bind to the promoter region of *glutathione S-transferase pi 1* (*GSTP1*) gene, increase DNA methylation levels of the region through recruiting DNA methyltransferase 1 (DNMT1), DNA methyltransferase 3A (DNMT3A) and DNA methyltransferase 3B (DNMT3B) into GSTP1 promoter region and diminish GSTP1 expression in ESCC cells (Chen J. L. et al., 2019). On the contrary, the demethylation of *GSTP1* via DNA methyltransferase inhibitor 5-Aza-CdR could weaken 5-FU resistance in LINC01419 overexpressed ESCC cells, demonstrating that LINC01419 functions as a modulator of 5-FU-based chemotherapy sensitivity in ESCC (Chen J. L. et al., 2019). Recently, oncogenic lncRNA HOTAIR has also been found to accelerate 5-FU resistance in esophageal cancer cells by promoting the promoter hypermethylation of methylene tetrahydrofolate reductase (MTHFR) gene. Silencing of HOTAIR could promote the apoptosis induced by 5-FU and alleviate cell proliferation and MTHFR promoter methylation of esophageal cancer cells. Moreover, overexpression of MTHFR has been shown to reverse 5-FU resistance caused by HOTAIR overexpression. Meanwhile, xenografts from HOTAIR-silenced esophageal cancer cells in nude mice also demonstrated the diminished 5-FU resistance, indicating that HOTAIR may represent a novel potential target for conquering 5-FU resistance of esophageal cancer (Zhang et al., 2020c). In addition, oncogenic lncRNA LINC01270 has also been shown to promote the resistance to 5-FU through regulating GSTP1 promoter methylation via recruiting three important DNA methyltransferases, including DNMT3A, DNMT3B, and DNMT1 (Li et al., 2021).

**TABLE 5:** Long non-coding RNAs (lncRNAs) and 5-FU resistance in esophageal cancer.

**LncRNAs**	**Expression^a^**	**Genes and Pathways**	**Drugs**	**References**
LINC01419	↑	GSTP1	5-FU	Chen J. L. et al., 2019
HOTAIR	↑	MTHFR	5-FU	Zhang et al., 2020c
LINC01270	↑	DNMT3A, DNMT3B, DNMT1	5-FU	Li et al., 2021
TP73-AS1	↑	–	5-FU	Zang et al., 2016
TUSC7	↓	miR-224/DESC1/EGFR/AKT	5-FU	Chang et al., 2018
LINC00261	↓	DPYD	5-FU	Lin K. et al., 2019

*^*a*^lncRNAs either up-regulated (↑) or down-regulated (↓) in 5-FU resistant esophageal cancer cells. This table shows six lncRNAs whose expression levels and underlying pathways in 5-FU resistance of esophageal cancer.*

By contrast, tumor suppressor lncRNAs play opposite role in development of 5-FU resistance of esophageal cancer. For instance, through suppressing miR-224 and regulating the ESC1/EGFR/AKT signaling, lncRNA TUSC7 has been demonstrated to conquer 5-FU resistance and increase the apoptosis of ESCC cells. Moreover, exogenous expression of DESC1 could enhance the sensitivity to 5-FU in ESCC cells (Chang et al., 2018). In addition, tumor suppressor lncRNA LINC00261 could also reverse the chemoresistance to 5-FU in human esophageal cancer cells through regulating DNA methylation-dependent expression inhibition of dihydropyrimidine dehydrogenase (DYPD). Exogenous expression of LINC00261 could significantly suppress cell growth and potentiate apoptosis sensitivity to 5-FU in ESCC cells. On the contrary, inhibition of LINC00261 has been shown to promote proliferation and apoptosis resistance of ESCC cells. Moreover, 5-aza-2′-deoxycytidine, a demethylation reagent, could reverse DNA methylation of DYPD promoter and DYPD activity in 5-FU resistant ESCC cells (Lin K. et al., 2019).

### Long Non-coding RNAs and Resistance to Other Drugs

Paclitaxel, adriamycin, and gefitinib are also used in esophageal cancer treatments. It has been found that multiple oncogenic lncRNAs participate in the resistance to these anti-cancer agents (Table 6). LncRNA DDX11-AS1, a highly expressed lncRNA in esophageal cancer tissues, has been found to increase paclitaxel resistance of esophageal cancer cells. Through binding to transcription factor TATA-box binding protein-associated factor 1 (TAF1) and up-regulating TAF1 expression, lncRNA DDX11-AS1 could promote the transcription of topoisomerase alpha 2 (TOP2A) and subsequently, increase TOP2A expression levels (Zhang S. et al., 2019). Silencing of lncRNA DDX11-AS1 could potentiate the inhibitory effects of paclitaxel on esophageal cancer xenografts in nude mice and suppress TOP2A expression, suggesting that lncRNA DDX11-AS1 may be a promising potential target for overcoming paclitaxel resistance of esophageal cancer (Zhang S. et al., 2019). LncRNA VLDLR was up-regulated in ESCC tissue and could promote adriamycin resistance of esophageal cancer cells via increasing ABCG2 expression (Chen Y. et al., 2019). LncRNA prostate androgen-regulated transcript 1 (PART1) has been found to confer the resistance to gefitinib, an oral epidermal growth factor receptor tyrosine kinase inhibitor (EGFR-TKI), through regulating miR-129/Bcl-2 pathway in ESCC cells. Interestingly, extracellular lncRNA PART1 could be secreted with exosomes, transferred to the sensitive ESCC cells, and promoted gefitinib resistance of ESCC cells. In addition, high expression of serum lncRNA PART1 in exosome was also associated with unfavorable response to gefitinib in ESCC patients (Kang et al., 2018). Through modulating PI3K-AKT-mTOR signaling, Linc01014 overexpression could also dramatically suppress the apoptosis of esophagus cancer cells and promote gefitinib resistance (Fu et al., 2020).

**TABLE 6:** Long non-coding RNAs (lncRNAs) and resistance to other drugs in esophageal cancer.

**LncRNAs**	**Expression^a^**	**Genes and pathways**	**Drugs**	**References**
DDX11-AS1	↑	TAF1/TOP2A	Paclitaxel	Zhang S. et al., 2019
VLDLR	↑	ABCG2	Adriamycin	Chen Y. et al., 2019
PART1	↑	miR-129/Bcl-2	Gefitinib	Kang et al., 2018
Linc01014	↑	PI3K-AKT-mTOR	Gefitinib	Fu et al., 2020

*^*a*^lncRNAs up-regulated (↑) in other drugs resistant esophageal cancer cells. This table shows four lncRNAs whose expression levels and underlying pathways in other drugs’ resistance of esophageal cancer.*

## Circular RNAs and Drug Resistance in Esophageal Cancer

Circular RNAs, a special type of endogenous circular ncRNAs, are generated through the process called back-splicing of linear precursor mRNA (pre-mRNA) transcripts and lack 3′ poly (A) tail and 5′ cap (Chen, 2016). CircRNAs could regulate gene expression through sponge adsorption of miRNA, modification of parental genes, and regulation of transcription and splicing of target genes. Amounting evidence has demonstrated that circRNAs are involved in multiple cellular processes and several malignancies, including esophageal cancer (Hu X. et al., 2019; Kristensen et al., 2019). Interestingly, circRNAs could not only serve as diagnostic and prognosis markers of esophageal cancer, but also participate in drug resistance (Zhang et al., 2020d; Zou et al., 2020). For instance, circRNA_001275, an up-regulated circRNA in DDP-resistant esophageal cancer cells and tissues, has been shown to accelerate cell growth and reduce the apoptosis of DDP-resistant cells. On the contrary, knockdown of circRNA_001275 inhibited the proliferation of DDP-resistant cells. It has been found that circRNA_001275 could contribute to DDP resistance in esophageal cancer through directly binding to and competitively sponging miR-370-3p to up-regulate Wnt family member 7A (Wnt7a) expression (Zou et al., 2020). In addition, via regulating miR-194-5p/JMJD1C axis, oncogenic circ_0006168 has been shown to potentiate Taxol resistance in ESCC (Qu et al., 2021). Recently, tumor suppressive circPSMC3, down-regulated in ESCC tissues and gefitinib-resistant (GR) ESCC cells, its overexpression could conquer gefitinib resistance, increase apoptosis rate and cleaved caspase-3 level in GR ESCC cells through modulating the miR-10a-5p/PTEN axis, which provide a promising therapeutic strategy for overcoming gefitinib resistance in ESCC (Zhu H. et al., 2021).

## Conclusion

**FIGURE 2:**
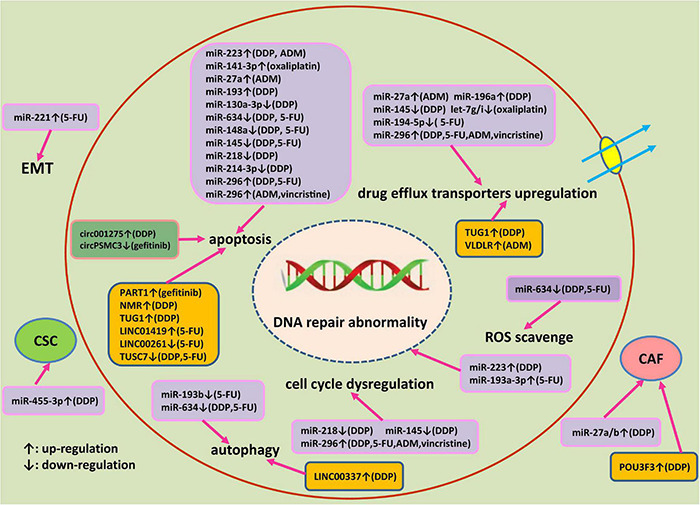
An outlined diagram of miRNAs, lncRNAs, and circRNA involved in the drug resistance of esophageal cancer. Multiple miRNAs, lncRNAs, and circRNA have been found to be linked to the drug resistance of esophageal cancer through altering cell proliferation, apoptosis, DNA damage repair, cell cycle progression, autophagy, cancer stem cell, and epithelial-mesenchymal transition via modulating corresponding target genes and signaling pathway.

Accumulating evidences have shown that ncRNAs significantly contribute to drug resistance of esophageal cancer. The corresponding mechanisms of miRNAs, lncRNAs, and circRNAs involved in drug resistance of esophageal cancer are illustrated in Figure 2. Multiple mechanisms including abnormal histone and DNA modifications, genomic amplification/loss and post-transcriptional regulations are involved in the dysregulation of 3 kinds of ncRNAs in esophageal cancer. Personalized therapy according to abnormally expressed miRNAs, lncRNAs, and circRNAs may be a promising way to overcome drug resistance. Silencing of oncogenic ncRNAs using small interfering RNAs (siRNAs) or short hairpin RNAs (shRNAs) have been reported to be effective in restoring the therapeutic sensitivity of esophageal cancer (Hu X. et al., 2019). Alternatively, ectopic expression of tumor suppressor ncRNAs have been demonstrated to be beneficial to conquer therapeutic resistance in esophageal cancer. Locked nucleic acid (LNA) modifications of ncRNAs can enhance *in vivo* stability and affinity. However, drug safety, immune-related toxicities or other adverse effects remain important issues to be solved for ncRNAs-based therapeutics. After searching the http://clinicaltrials.gov database, we found that there are still no therapeutic clinical trials based on ncRNAs in esophageal cancer currently. Due to the complexities of cancer signaling pathways, the inhibition of a single target signaling or ncRNA may show minor effects. The combination based on modulation of ncRNAs expression and classical chemotherapy, novel targeted therapy or immunotherapy may be a promising choice to treat advanced or metastatic esophageal cancer patients. However, selecting key target ncRNA from numerous candidate ncRNAs for the intervention remains a difficult issue.

## Author Contributions

LW and MY conceived the review, acquired data, provided project funding, and drafted the manuscript. MY and ZL reviewed and supervised the manuscript. JS, NZ, YS, and TW undertook the initial research. All authors read and approved the submitted version.

## Conflict of Interest

The authors declare that the research was conducted in the absence of any commercial or financial relationships that could be construed as a potential conflict of interest.

## Publisher’s Note

All claims expressed in this article are solely those of the authors and do not necessarily represent those of their affiliated organizations, or those of the publisher, the editors and the reviewers. Any product that may be evaluated in this article, or claim that may be made by its manufacturer, is not guaranteed or endorsed by the publisher.
